# Growing at the right time: interconnecting the TOR pathway with photoperiod and circadian regulation

**DOI:** 10.1093/jxb/erac279

**Published:** 2022-06-23

**Authors:** Reynel Urrea-Castellanos, Camila Caldana, Rossana Henriques

**Affiliations:** Max Planck Institute of Molecular Plant Physiology, Am Mühlenberg, Potsdam-Golm, Germany; Max Planck Institute of Molecular Plant Physiology, Am Mühlenberg, Potsdam-Golm, Germany; School of Biological, Earth and Environmental Sciences, University College Cork, Cork, Ireland; Environmental Research Institute, Cork, Ireland; Consejo Superior de Investigaciones Científicas, Spain

**Keywords:** Carbon partitioning, circadian clock, plant growth, photoperiod, starch metabolism, TOR pathway

## Abstract

Plants can adjust their growth to specific times of the day and season. Different photoperiods result in distinct growth patterns, which correlate with specific carbon-partitioning strategies in source (leaves) and sink (roots) organs. Therefore, external cues such as light, day length, and temperature need to be integrated with intracellular processes controlling overall carbon availability and anabolism. The target of rapamycin (TOR) pathway is a signalling hub where environmental signals, circadian information, and metabolic processes converge to regulate plant growth. TOR complex mutants display altered patterns of root growth and starch levels. Moreover, depletion of TOR or reduction in cellular energy levels affect the pace of the clock by extending the period length, suggesting that this pathway could participate in circadian metabolic entrainment. However, this seems to be a mutual interaction, since the TOR pathway components are also under circadian regulation. These results strengthen the role of this signalling pathway as a master sensor of metabolic status, integrating day length and circadian cues to control anabolic processes in the cell, thus promoting plant growth and development. Expanding this knowledge from *Arabidopsis thaliana* to crops will improve our understanding of the molecular links connecting environmental perception and growth regulation under field conditions.

## Teaser

To ensure growth responses accurately match the available energy resources, plants integrate external cues (e.g. photoperiod) with metabolic signals (carbon/energy) to coordinate the diel activity of the TOR signalling pathway.

## Introduction

Plants have adapted to the conditions imposed by two types of the Earth’s motion, rotation and revolution. These planetary events impose a ~24 h diel cycle of light and dark periods, the duration of which changes according to the time of the year and latitude. Thus, sun radiation (light) and the 24 h diel cycle represent two key environmental conditions to which plants must adapt in order to grow. To use efficiently the energy provided by the sun, plants are capable of sensing and responding to light signals, and for this purpose they harbour light receptors that mediate the up-regulation of processes that should take place during the day and not at night, such as photosynthesis (Yang and Liu, 2020). Likewise, to synchronize biological processes to the light/dark cycles, plants evolved an internal biological timekeeper paced to the 24 h diurnal cycle, referred to as the circadian clock (de Montaigu *et al.*, 2010). The clock comprises an intricate network of regulators where several interconnected loops of transcriptional and post-translational events ensure adequate coordination between external (light, temperature) and internal (rhythmic accumulation of regulators) cues, allowing plants to adjust their growth patterns to specific times of the day and the year (Henriques *et al.*, 2018).

**Fig. 1.**
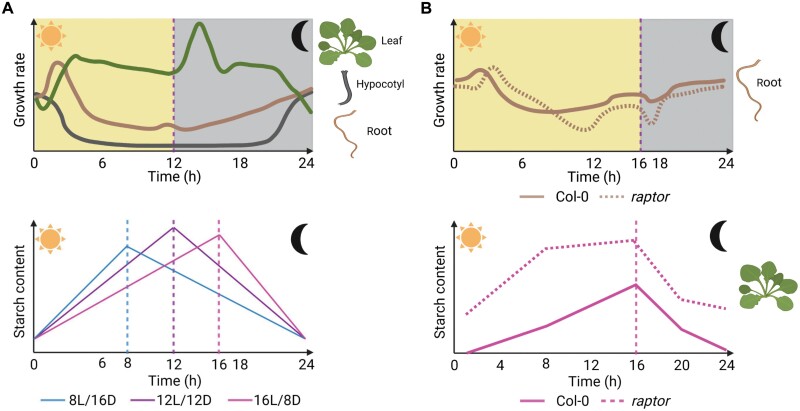
Diel patterns of organ growth and management of carbon (C) resources are modulated by the photoperiod, the clock, and the target of rapamycin complex (TORC). (A) Upper panel: Plant organs grow at a different pace along the 24 h diel cycle. The relative expansion rate of rosette (green), root extension rate (brown), and hypocotyl elongation rate (grey) are depicted for Arabidopsis Col-0 plants grown under 12 h light/12 h dark conditions. Growth profiles are based on Apelt *et al.* (2017), Yazdanbakhsh *et al.* (2011), and Nusinow *et al.* (2011), respectively. In the light period, C resources and light signals promote growth in the early morning in both leaf and root organs. In hypocotyls, however, light strongly inhibits elongation via the degradation of PIFs. During the remainder of the light period, rosettes maintain a steady rate of extension, whereas root growth declines. During the night, rosettes, roots, and hypocotyls differ in their growth patterns. Whereas the rosette extension rate peaks 2 h after dusk and then declines, root growth increases steadily during the night, while hypocotyl elongation occurs in the second half of the dark period. These diel growth patterns display a similar trend both under short and long photoperiods, although with some specific variations (see main text for details). Lower panel: When C partitioning is considered, during the light period photosynthates are partitioned towards sugars or stored in the leaves as starch. This starch will be used at night to fuel growth and maintain metabolism at the whole-plant level. Consequently, starch accumulates during the light period and is broken down during the dark period. Arabidopsis is an annual species under selective pressure to optimize C usage (Scialdone and Howard, 2015) and it will rely on the circadian clock and light signalling pathways to coordinate starch turnover with photoperiod (data modified from Seki *et al.*, 2017). Therefore, as the length of the light period shortens, the rate of starch synthesis increases. In contrast, as the length of the night increases, the rate of starch breakdown slows down to ensure that there are enough C resources for the long night. (B) Plants possess sensors such as the TORC, which is able to integrate external signals with the internal energy status of the plant to coordinate growth. The TORC is activated by light and sugars to mediate anabolic growth processes. Disruption of TORC function is known to impair growth in leaves, hypocotyls, and roots. Upper panel: When the root extension patterns of Col-0 and *raptor1B* (*raptor*) mutants grown under long photoperiods were compared, the mutant showed a delay in the early morning peak and root extension was reduced, particularly in the second part of the light period and shortly after dusk (modified from (Salem *et al.*, 2018). Lower panel: Along with this impaired growth phenotype, *raptor* mutants exhibited an excess of starch accumulation in both light and dark periods under long-day conditions, suggesting a deficiency in C resource management (modified from Salem *et al.*, 2018). Image created with BioRender.com.

Plant growth depends on light to assimilate carbon (C) through photosynthesis in the leaves; however, the manner by which the photosynthates are used and allocated will change according to the plant’s life strategy and physiology. In this review, we will focus on *Arabidopsis thaliana* (Arabidopsis), an annual species that is under strong selective pressure to optimize C usage and maximize seed production (Scialdone and Howard, 2015). The photosynthates of Arabidopsis are either partitioned into sugars, which can be used immediately to fulfil metabolism and growth, or stored as starch in source leaves (Smith and Stitt, 2007). Most of these photosynthates can also be translocated into non-photosynthetic sink tissues (e.g. roots and young leaves) to sustain their metabolic needs and growth (Roberts *et al.*, 1997; Imlau *et al.*, 1999; Sonnewald and Fernie, 2018). At night, the C stored in the form of starch can be remobilized to generate energy and support structural biomass (Stitt and Zeeman, 2012). Accordingly, as an emerging property of C resource synthesis and utilization at the whole-plant level, the rate of starch accumulation is almost linear in the light period and then decreases steadily during the night to ensure that cellular metabolism is maintained during the dark period (Fig. 1A, lower panel). This pattern will be adjusted to specific photoperiods due to the concerted action of the circadian clock and light signalling pathways, which ensure the optimal use of C resources throughout the diel cycle (Smith and Stitt, 2007).

In addition to a proper running clock and adequate light perception, plants also require cellular energy sensors that help integrate external signals with the energy status (C availability) and promote the efficient use of C resources. One of these sensors is the target of rapamycin (TOR), a master kinase that associates with RAPTOR (Regulatory-associated protein of TOR) and LST8 (Lethal with SEC13 protein 8) to form the TORC complex. Animals and yeast also possess an additional complex, referred as TORC2 (Saxton and Sabatini, 2017), which lacks RAPTOR but includes LST8 and other TOR interacting proteins for which no homologues have been found in plants (Dobrenel *et al.*, 2016). In the plant cell, TORC controls a range of anabolic processes, including ribosome biogenesis, the synthesis of proteins, lipids, amino acids, and nucleotides, and cell proliferation throughout plant growth and development, which are intensively covered in excellent reviews (Henriques *et al.*, 2014; Dobrenel *et al.*, 2016; Schepetilnikov and Ryabova, 2018; Brunkard, 2020; Artins and Caldana, 2022). In this review, we will discuss how light and the circadian clock shape the patterns of growth of different plant organs in coordination with energy sensors, such as TORC, and overall resource availability during the diel cycle in Arabidopsis.

## Integration of C resource availability with light signals and circadian perception regulates plant growth

Advances in high-throughput imaging techniques allowed a deeper and more accurate characterization of the diel patterns of growth of different plant organs (Yazdanbakhsh *et al.*, 2011; Apelt *et al*., 2015, 2017). These techniques use non-invasive imaging platforms that automatically capture, in short time lapses, the growth of a given organ and allow sequential image processing to calculate extension rates, among other parameters. For a better understanding of the current plant growth imaging technologies, we recommend the recent review by Olas *et al.* (2020). These different techniques assess organ expansion, which is a consequence of two interlocking processes: an increase in cell number and an increase in cell size. The latter process is triggered by the synthesis of cellular building blocks (e.g. cell wall and protein synthesis) coupled with changes in cell wall extensibility and water uptake, which will result in a gain of biomass and volume, respectively, over time (Verbančič *et al.*, 2018; Hilty *et al.*, 2021). In the following sections we describe the diel growth patterns of different organs, which highlight their diverse dynamics in the model dicot species Arabidopsis. However, it is important to mention that the environmental control of these growth patterns differs among plants. In monocots, leaf extension responds more strongly to temperature variations and the photoperiod-dependent effect is less pronounced than in dicots, thus indicating that factors influencing growth dynamics can differ between the two groups of flowering plants; we advise the readers to consult other reviews for further insights into this differential regulation (Poiré *et al.*, 2010; Atkinson *et al.*, 2016).

## Growth regulation in leaves

In Arabidopsis soil-grown plants (between 17 d and 24 d after sowing), the onset of light promotes a rapid expansion in leaves, reaching the maximum at 3–4 h after dawn. After this point, leaf expansion slightly decreases for the rest of the light period when plants are grown in soil under 12 h light/12 h dark (12L/D) conditions (Apelt *et al.*, 2017). During the dark period, leaf expansion peaks 2 h after dusk and this peak is followed by a decrease for the rest of the night (Fig. 1A, upper panel). Changes in the photoperiod will slightly affect the amplitude of these diel peaks, although they share a similar trend. Under long days (LDs; 16 h light/8 h dark), the expansion peak after dusk is shifted back 1 h, while the expansion rate of rosettes growing under short days (SDs; 8 h light/16 h dark) does not decrease greatly after the peak of growth around dusk. This diel rosette expansion pattern is maintained in other Arabidopsis accessions (e.g. Col-4 and WS-2), with the only exception being the larger amplitude of the morning peak under 12L/D or a 3 h earlier shift under LD conditions in WS2. The initial burst of growth is thought to be driven by photoassimilates as a response to the onset of light after a dark period, and it is abolished when plants are transferred to continuous light (LL) (Apelt *et al.*, 2017). Furthermore, when measuring individual leaf elongation rates, a reduction in light intensity also diminished the peak of growth 2 h after dawn (Dornbusch *et al.*, 2014), indicating that C availability driven by photosynthesis would account for the amplitude of leaf growth at the beginning of the day. Subsequently, in the second part of the light period, photosynthesis provides the C supply needed to keep a stable rosette expansion rate until the onset of night. Interestingly, rosette expansion was stimulated in the first hours of the dark period in all photoperiods. It is possible that this burst of growth is associated with high sugar availability due to the combined action of starch breakdown and the photosassimilates remaining from the light period (Apelt *et al.*, 2017). Overall C management and growth dynamics in rosettes are flexible enough to adapt to transient (e.g. cloudy days) or long-term (e.g. photoperiod) changes in environmental cues, so that growth responses are optimized at the whole-plant level. For instance, in SD conditions, net growth is higher at night and is proposed to be restricted during the light period (Sulpice *et al.*, 2014; Apelt *et al.*, 2017; Mengin *et al.*, 2017), suggesting that more C is being partitioned towards storage as starch to sustain growth in the long night (Sulpice *et al.*, 2014; Kölling *et al.*, 2015; Mengin *et al.*, 2017). On the other hand, in longer photoperiods more of the fixed C is allocated to growth, leading to higher net growth during the light period (Mengin *et al.*, 2017). Therefore, C deposition into structural biomass would occur preferentially during the dark period under SDs, whereas under LDs it would take place primarily in the light period (Apelt *et al.*, 2017).

This dynamic management of C resources is also under circadian regulation to ensure optimal growth responses in Arabidopsis. Net growth (Graf *et al.*, 2010) and rosette expansion rates (Apelt *et al.*, 2017) are reduced in circadian clock mutants with either shorter or longer internal periods in comparison to the wild type (Dodd *et al.*, 2005). This growth impairment could be a consequence of premature starch exhaustion at night in short-period clock mutants (e.g. *lhy/cca1*) or due to suboptimal balance between C assimilation, storage, and utilization in the case of long-period mutants (e.g. *ztl-1* or *prr7prr9*) (Graf *et al.*, 2010; Apelt *et al.*, 2017). Furthermore, a closer look at the effect of clock mutations on the diel expansion patterns of rosettes and leaves reveals that growth peaks are either shifted or reduced in amplitude under 12L/D when compared with the wild type, suggesting the importance of the clock in coordinating growth and C management at the transition between light and dark periods. Indeed, C partitioning in *lhy/cca1* and *prr7/prr9* clock mutants is altered (Kölling *et al.*, 2015). While for the wild type C flux towards starch increased after the onset of light and reached a peak 6 h later, in the *lhy/cca1* mutant this peak occurred earlier, at 2 h, and then C deposition decreased steadily. By contrast, for the *prr7/prr9* mutant, channelling of C towards starch remained almost stable throughout the light period. Furthermore, circadian regulation of gene expression could also influence C management and partitioning in leaves, given that transcript levels of the sugar transporters *AtSWEET11*, *AtSWEET12*, and *AtSUC2* were shown to oscillate, with the highest expression levels being at night (Haydon *et al.*, 2011; Durand *et al.*, 2018). Overall, the circadian clock would ensure the coordination of starch turnover and sugar transport along the diel cycle to avoid periods of starvation when environmental cues restrict the C supply from photosynthesis (Smith and Stitt, 2007; Webb *et al.*, 2019).

## Growth regulation in roots

As sink organs, roots display a distinct diel pattern of growth, since they rely on C reallocation from source tissues (leaves) (Yazdanbakhsh and Fisahn, 2011). In Arabidopsis seedlings grown *in vitro* in solid medium for 11 d under 12L/D conditions, the root extension rate peaks between 1 h and 2 h after the onset of light and then decreases for the rest of the light period (Yazdanbakhsh *et al.*, 2011). After dusk, root extension increases steadily throughout the night (Fig. 1A, upper panel). This pattern is mostly maintained under short and long photoperiods, although in the latter, the root extension rate begins to increase 4 h before dusk (see discussion below). Moreover, similarly to rosette expansion and leaf elongation, the initial burst of root growth occurs in the first part of the day (Yazdanbakhsh *et al.*, 2011). This supports the view that exposure to light triggers a rapid increase in photoassimilates in source leaves, which will be preferentially exported to sink organs to sustain metabolism and growth. After the peak of growth in the morning, root elongation strongly decreases and remains stable for the rest of the light period, probably as a result of the allocation of C into different pools rather than solely to the sink organs (e.g. storage in the form of starch). Indeed, it was shown that during the first part of the day under 12L/D, approximately 20% of the fixed C was allocated to starch; however, from 6 h onwards [*zeitgeber* time (ZT)6], this increased to 40%, indicating a reduction of sugars being exported out of the source leaves in the second part of the day (Kölling *et al.*, 2015). Interestingly, as mentioned above, root extension begins to increase in the light from ZT12 to ZT16 under LD, but not in SD or 12L/D conditions (Yazdanbakhsh *et al.*, 2011). This observation would be in agreement with the higher availability and export of fixed C towards sink organs during the second part of the day in long photoperiods, since C availability is relaxed and starch is already beginning to be degraded towards the end of the light period (ZT14) (Fernandez *et al.*, 2017). At the beginning of the night, however, starch breakdown in source leaves will provide C to sustain metabolism and growth in sink organs. Consequently, the rate of root elongation during the night is heavily compromised in mutants with defects in starch turnover; although this phenotype can be partially reverted by the addition of exogenous sugars to the growth medium (Yazdanbakhsh *et al.*, 2011).

Similar to leaf expansion, root extension at night strongly relies on C resource management, which is under circadian control in Arabidopsis. For instance, under LL or continuous darkness, diel root growth oscillations were maintained in the subjective light and dark periods (Yazdanbakhsh *et al.*, 2011). Furthermore, root growth patterns were strongly affected in mutants of the evening complex (EC) (*elf3* or *elf4*) and morning loop components (double mutant *cca1/lhy*) (Yazdanbakhsh *et al.*, 2011). Confirming its premature starch exhaustion, in *cca1/lhy* the root extension rate was affected in the last hours of the night, when less C is available to fuel growth (Lu *et al.*, 2005; Graf *et al.*, 2010). By contrast, in *elf3* mutants, changes in growth dynamics were not associated with C availability at night, given that these plants accumulate more starch and sugars compared with the wild type, but instead, they could be a consequence of growth derepression in the light period that affected specifically the root clock (similar to the role of *ELF3* in hypocotyl growth; see below) (Yazdanbakhsh *et al.*, 2011). Overall, root growth relies on C import from leaves and the management of C reserves during the light period to sustain root expansion at night, and the circadian clock coordinates the use of available resources to avoid periods of starvation.

## Growth regulation in hypocotyl

In contrast to leaves and roots, in 10-day-old Arabidopsis seedlings grown in solid medium under 12L/D conditions, hypocotyl elongation decreases rapidly at the beginning of the day and remains absent for the rest of the light period. However, similar to roots, hypocotyls elongate during the night under 12L/D conditions, reaching a peak of growth at the end of the dark period (Fig. 1A, upper panel). C availability strongly regulates hypocotyl elongation, as exogenous sugar supply can offset the inhibitory effect of light on hypocotyl growth (Stewart *et al.*, 2011; Kelly *et al.*, 2021) and enhance the elongation of this organ in response to high temperatures (Hwang *et al.*, 2019). At the molecular level, light-mediated hypocotyl inhibition relies on phytochrome B-dependent degradation of Phytochrome Interacting Factors (PIF3, 4, and 5), which promote hypocotyl growth (Nozue *et al.*, 2007; Lorrain *et al.*, 2008; Soy *et al.*, 2012). In addition, the Pseudo Response Regulators (PRR7, PRR9), which are components of the core oscillator, regulate PIF transcriptional activity due to their ability to heterodimerize with and prevent PIF3 binding to its target promoters under SD conditions (Martín *et al.*, 2018). Hypocotyl elongation peaks at subjective night in seedlings grown under LL, suggesting the involvement of the EC in this process (Niwa *et al.*, 2009). The EC (ELF3, ELF4, and LUX) coordinates the transcription of several genes involved in temperature response, photosynthesis, growth, and phytohormone signalling, especially at the end of the dark period (Ezer *et al.*, 2017). The EC inhibits PIF expression at dusk and during the first part of the night (Nusinow *et al.*, 2011). Therefore, the combined action of PRRs and the EC ensures that the accumulation of PIFs is restricted to the dark period when they promote hypocotyl elongation, mostly at the end of the night. This response seems to increase non-linearly with the length of the dark period, further strengthening the relevance of the clock in regulating the accumulation and transcriptional activity of PIFs (Niwa *et al.*, 2009).

Together, these findings highlight the role of the light signalling pathway and the circadian clock in ensuring that the growth rate of each organ, at a given developmental stage, is well aligned with the diurnal cycle and C availability to allow the most efficient use of resources at the whole-plant level.

## TORC integrates metabolic and environmental signals to regulate growth at specific times of the day

The TORC is one of the major energy sensors in plants and is required to promote growth in different organs along their life cycle (Caldana *et al.*, 2019). For instance, in Arabidopsis, shoot and root apical meristems require TORC to activate cell proliferation in response to light and sugar signals (Li *et al.*, 2017). Furthermore, sugar transport through plasmodesmata between source and sink leaves is also regulated by TORC (Brunkard *et al.*, 2020); in agreement with this hypothesis, *lst8* mutants were shown to be affected in their shoot-to-root phloem trafficking (Moreau *et al.*, 2012). This accumulated evidence strengthens the notion that TORC is involved in long-distant transport and sugar sensing in both sink and source organs in Arabidopsis. Possibly, TORC could link central metabolism and biosynthetic processes to coordinate growth responses according to C/energy availability (Xiong *et al.*, 2013; Zhang *et al.*, 2016). Moreover, inhibition of TOR impaired the entrainment of the circadian clock by sugars, indicating that management of C resources mediated by TOR might also include a clock component (Zhang *et al.*, 2019) (see below).

The synthesis of structural components in the cell, such as proteins, occurs preferentially in the light period, when photosynthesis takes place and the plant can sustain high-energy-demanding processes (Sulpice *et al.*, 2014). TOR is known to promote the translation of ribosomal proteins (Deprost *et al.*, 2007; Schepetilnikov and Ryabova, 2018; Scarpin *et al.*, 2020) and therefore could function as a key mediator in converting photoassimilates (primarily sugars) into structural components in the cell. To ensure that growth responses occur at the most efficient time, diel sensing of C resources and their management should be coupled to TORC activity. Light-to-dark transitions trigger a remobilization of C resources, as described for starch dynamics, and TOR activity could be especially relevant at these times of the day to regulate the peaks of growth described previously for leaves and roots (see Fig. 1A, upper panel). Indeed, lack of *RAPTOR1B* affects the diel expansion pattern of primary roots under LD conditions (Salem *et al.*, 2018). *raptor1B* mutants show delayed root growth after the onset of light and a strong reduction shortly after dusk, suggesting a critical role for TORC during these transition periods (Fig. 1B, upper panel). Furthermore, from the second part of the light period (ZT9) onwards, the root elongation rate of *raptor1B* was consistently reduced. This overall root growth impairment, associated with specific metabolic changes, was also observed under TORC inhibition (Ren *et al.*, 2012; da Silva *et al.*, 2021). However, it remains to be elucidated if similar diel growth defects associated with the *raptor1b* mutation also occur in other plant organs, such as leaves.

**Fig. 2.**
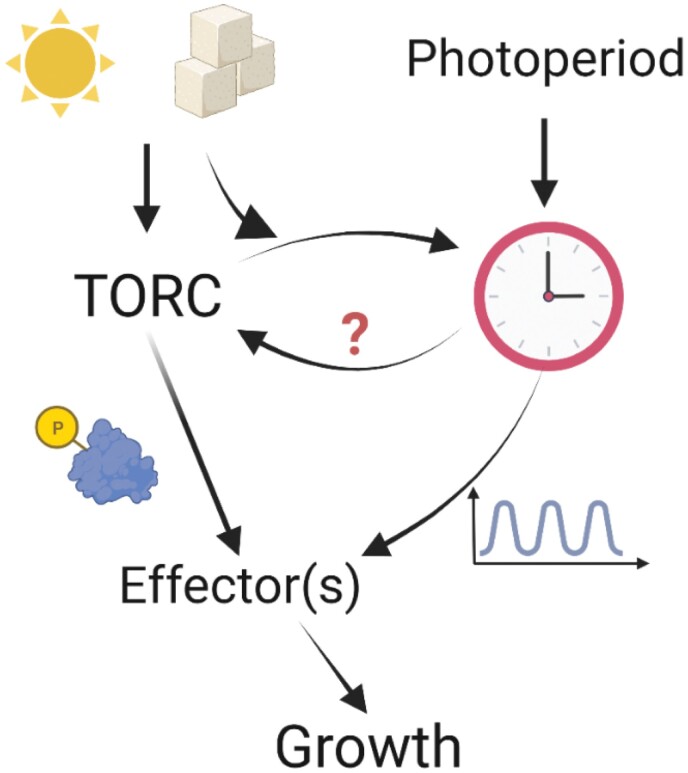
Working model connecting the target of rapamycin (TOR) pathway with the circadian clock to coordinate plant growth. Light and energy-status signals will activate the TOR complex (TORC) to phosphorylate downstream effector(s) (proteins) that promote growth. In parallel, gene expression and/or protein levels of these TORC effectors will be modulated on a daily and/or seasonal basis by the circadian clock. Thus, the interplay between the TOR pathway and the clock will determine the final growth responses promoted by these effectors. Moreover, sugar entrainment of the circadian clock requires TOR activity, suggesting that TOR could feed back to the core oscillator (Zhang *et al.*, 2019). However, the exact molecular mechanisms underlying the direct regulation between TORC components and the circadian clock have yet to be characterized. Image created with BioRender.com.

In Arabidopsis, the down-regulation of *TOR* gene expression (Caldana *et al.*, 2013) or the lack of TORC components (Moreau *et al.*, 2012; Salem *et al.*, 2018) led to the accumulation of starch during the day and night period, especially under LD conditions (Fig. 1B, lower panel). However, whether this excess of starch could modulate the circadian clock is unknown. High levels of starch were accompanied by the accumulation of triacylglycerides and the reduction of sucrose levels (Caldana *et al.*, 2013; Salem *et al.*, 2018). Therefore, TOR inhibition could reduce the amount of fixed C being used for anabolic growth processes, with more fixed C then being redirected towards storage compounds (e.g. starch or triacylglycerides) during the day. This shift towards storage would lead to overall low sucrose levels during the diel cycle, and consequently less available C to fuel growth. Still, it remains to be elucidated whether these metabolic phenotypes of TORC-affected mutants are due to (i) an inability to accurately sense the levels of internal sugars/energy, (ii) the direct regulation of pathways controlling sucrose and starch metabolism, or (iii) a combination of both processes to ensure energy homeostasis for growth. This daily mismanagement of resources would imply that the growth responses of mutants of the TORC, as well as *TOR* gene-silencing lines, would lag behind the wild type every day, similar to the *raptor1b* mutant. In agreement with this hypothesis, gibberellin-mediated diurnal leaf expansion was shown to require TOR to enhance the end-of-the-day expression of *GIBBERELLIN 3-BETA-DIOXYGENASE 1* (*GA3ox1*), an enzyme involved in the synthesis of bioactive gibberellins (GA_4_) and overall growth promotion (Prasetyaningrum *et al.*, 2021). This regulation of growth seems to extend to specific developmental transitions, since two independent mutants for *RAPTOR1B* displayed a delay in growth during the transitions from the juvenile to the adult vegetative stage, or from the adult to the reproductive stage, when energy requirements are extremely high (Salem *et al.*, 2018). From these findings, a potential model emerges in which the TORC would respond to light signals to activate anabolic processes, while managing C mobilization in coordination with the circadian clock to promote growth, especially at light-to-dark transition periods (Fig. 2).

## Interconnection between the TOR pathway and the circadian clock: mutual feedback or unidirectional regulation?

The findings described so far suggest that TORC could integrate light, the time of day, and C assimilate levels to ensure that growth occurs at the most advantageous time. This interplay would then require a specific coordination between the TOR pathway and the circadian clock. Such a relationship has been characterized in mammals (Khapre *et al*., 2014a, b; Lipton *et al.*, 2015; Wu *et al.*, 2019), and recent findings suggest this interconnection is also conserved in plants.

Although light and temperature are the main clock *zeitgebers*, accumulating evidence shows that sugars and metabolites are also involved in circadian entrainment (Dodd *et al.*, 2007; Haydon *et al.*, 2013). Early reports showed that over 40% of sucrose-, glucose-, and carbon fixation-responsive genes displayed circadian behaviour. Moreover, although some of these oscillations were maintained in the starch-less *pgm* (*phosphoglucomutase*) mutant, others increased in their amplitude, suggesting that mis-regulation of sugar levels in these plants (high in the day and very low in the night) impacts on circadian function (Bläsing *et al.*, 2005). The molecular mechanisms underlying these responses seem to depend on *PRR7*, a component of the central oscillator (Frank *et al.*, 2018). Under low-energy conditions, the expression of *PRR7* is up-regulated due to the direct binding of bZIP63 to its promoter, which will result in a dynamic adjustment of the circadian phase, allowing the clock to respond to the diel changes in sugar levels. bZIP63 activity is regulated by SnRK1, and is required to sustain adequate growth responses, especially under shorter photoperiods. In fact, both the rosette and leaf size of *bzip63* mutants are reduced under SDs, and this growth impairment is associated with a defect in cell growth and not in cell proliferation (Viana *et al.*, 2021). Interestingly, among the 60% of oscillating genes affected in *bzip63* plants is the *40S Ribosomal protein S6 Kinase 1* (*S6K1*), a conserved TOR target, which suggests an involvement of the TORC pathway in circadian-controlled growth responses. Moreover, recent findings show that the TOR pathway could actually regulate the pace of the clock (Zhang *et al.*, 2019; Wang *et al.*, 2020). Sugar deprivation, nicotinamide treatment, or TOR inhibition (by either amiRNA, RNAi strategies, or chemical inhibition using Torin 1) resulted in a similar increase in the circadian period. Moreover, the longer period observed upon sugar depletion was correlated with lower TOR activity (measured as S6K1 phosphorylation) and could be rescued by re-applying glucose. *TOR* inhibition by RNAi, however, disrupted this rescue effect and the circadian period length was still extended (Zhang *et al.*, 2019). Depletion of cellular energy levels either by nicotinamide treatment (which decreases the ATP concentration) or by disrupting the mitochondrial electron transport chain also resulted in an extended period (Zhang *et al.*, 2019; Wang *et al.*, 2020). Interestingly, root length and meristem activation were affected under TOR inhibition, further confirming the role of this pathway in regulating plant growth. Taken together, these results would suggest that TOR activity levels could feed back to the core oscillator and affect the pace of the clock.

However, this interaction seems to be reciprocal, since components of the TOR pathway are also under circadian regulation. PRRs were shown to mediate TOR signalling via the Tandem Zinc Finger 1 (TZF1) protein, an RNA-binding protein localized in cytoplasmic processing bodies (P-bodies) (Li *et al.*, 2019). PRR5, 7, and 9 could directly bind to the promoter region of *TZF1* in whole seedlings, although only PRR7 and 9 were shown to inhibit its transcription in leaf protoplasts (Wang *et al.*, 2020). *TZF1* and *TZF4* overexpression negatively regulated glucose-TOR signalling, possibly by binding *TOR* mRNA and triggering its degradation. These findings would indicate that PRRs could modulate glucose-TOR signalling via their negative regulation of *TZF1*. However, the exact mechanism for this is still not fully understood, since PRR regulation of TZF1 has been demonstrated in source organs such as leaves, and glucose-TOR signalling was shown to affect only the root meristem (sink organ). Moreover, how light signals perceived in the aerial part of the plant would be integrated into root clocks is another open question. Nevertheless, these findings suggest that clock components could modulate *TOR* transcript levels, even if indirectly.

There is growing evidence suggesting that the clock could regulate components of the TOR pathway at the post-translational level. It has been recently shown that specific post-translational modifications, such as protein phosphorylation, display circadian rhythms. In fact, most of these rhythmic phosphorylation events were shown to peak at subjective dawn [circadian time (CT)24] under LL conditions (Krahmer *et al.*, 2022). Interestingly, among the identified proteins displaying rhythmic accumulation and phosphorylation is the 40S Ribosomal Protein S6 (RPS6), a direct target of S6K1 and widely used read-out of TORC activity. Moreover, the authors proposed that S6K1, among other kinases, could be involved in this phospho-dawn mechanism, further confirming the role of the clock in modulating TOR pathway activity. These results confirm earlier reports describing diel and circadian phosphorylation of RPS6 (Enganti *et al.*, 2018). Under LDs, RPS6 phosphorylation (RPS6-P) peaks at early morning and is minimal before dawn. However, under LL, this pattern was reversed, with RPS6-P being maximal at the end of the subjective night (dawn). These findings lead the authors to hypothesize that both light signalling and the circadian clock modulate the amplitude of RPS6 phosphorylation in an opposite manner. Interestingly, although these oscillatory patterns were maintained under 0% and 1% sucrose treatments, at 3% sucrose RPS6-P was affected, suggesting that high sugar concentrations disrupt both light and clock signal integration. RPS6-P associated with large polysomes at early morning under LD conditions, but under LL this association was shifted to the end of subjective night. These findings indicate that light and circadian regulation of RPS6-P seemed to occur with polysomal association or that this regulation allows RPS6 interaction with polysomes. Considering the evolutionary conservation of RPS6-P, the identification of this conflicting regulation between light signals and the circadian clock could have a broader impact on the environmental regulation of plant growth. In order to dissect this possibility, Panchy *et al.* (2020) created a mathematical model in which light and dark signals would act independently of and oppositely to clock signals in the regulation of RPS6-P. These inputs would be integrated into a feed-forward circuit, which predicted a peak of RPS6-P at early morning. Interestingly, this peak would respond to changes in day length occurring at either dawn or dusk the previous day, suggesting that an anticipatory mechanism is in place. Incorporation of real day-length data from two locations at different latitudes (Cape Verde and Norway) further confirmed the predictive role of this early morning peak of RPS6-P in seasonal perception. Transient changes, such as fluctuating photosynthetic radiation from nearby vegetation or a passing cloud, could also be perceived by the model, indicating that it could maintain a certain robustness to rapid weather fluctuations while keeping the ability to measure day-length changes. Expanding this clock+light early morning regulation to circadian genes (e.g. peaking at early morning under LD, or at subjective night under LL) revealed an enrichment in the Gene Ontology categories of ‘light responses’, ‘photosynthetic regulation’, and ‘chloroplast localization’, suggesting an association between early morning peak adjustment and these metabolic functions. These processes could then add to the anticipatory function of the circadian clock in generating external coincidence mechanisms, which include (i) the timing of maximal hypocotyl elongation (Nozue *et al.*, 2007) and (ii) photoperiod-dependent induction of flowering time (Andrés and Coupland, 2012). Therefore, early morning RPS6-P responsiveness to changes in day length could be part of a molecular link, which would also include metabolic regulatory processes to ensure that plant growth responses are adjusted to specific seasons.

## Conclusions

New technologies to determine diel patterns of shoot and root growth resulted in the detailed characterization of the photoperiod and circadian regulation of plant growth responses. These findings, combined with metabolic profiling experiments, highlighted the relevance of coordinating C availability in sink and source organs to ensure the optimal timing of growth. Molecular, genetic, and physiological data place the TORC at the centre of this network. However, evidence is still lacking on the dynamics of accumulation of TORC components, especially at the protein level. Although great progress has been made to identify and test TOR inhibitors, full characterization of these proteins and their associated regulatory networks has been hampered by the lack of specific antibodies. Nevertheless, innovative strategies combining wet lab data with *in silico* analysis and mathematical modelling revealed how early morning RPS6-P could track day-length changes, especially at higher latitudes, improving our understanding of the association between TOR signalling, circadian function, light perception, and seasonality (Fig. 2). However, mapping the molecular link between TOR signalling and environmental regulation outside controlled laboratory conditions is still one of the main challenges in the field. Another challenge is to characterize the TOR pathway in crop species. Accumulation of AtTOR ectopically expressed in rice was shown to provide tolerance to abiotic stress while maintaining yield (Bakshi *et al.*, 2017), further confirming the potential of the TOR pathway in promoting plant growth responses under detrimental conditions. Considering the challenges climate change will pose for agricultural systems, associated with an increased awareness of the need for sustainable practices, improving our basic understanding of a major growth-regulatory signalling pathway could improve the molecular toolkit available to generate climate-proof species that are able to maintain their yield and productivity under a changing environment.
